# Host DNA released by NETosis in neutrophils exposed to seasonal H1N1 and highly pathogenic H5N1 influenza viruses

**DOI:** 10.1186/s12931-020-01425-w

**Published:** 2020-06-23

**Authors:** Louisa L. Y. Chan, John M. Nicholls, J. S. Malik Peiris, Yu Lung Lau, Michael C. W. Chan, Renee W. Y. Chan

**Affiliations:** 1 CUHK-UMCU Joint Research Laboratory of Respiratory Virus & Immunobiology, Hong Kong, China; 2 Department of Paediatrics, Faculty of Medicine, The Chinese University of Hong Kong, Hong Kong, SAR China; 3 Lee Kong Chian School of Medicine, Nanyang Technological University, Singapore, Singapore; 4 School of Public Health, Hong Kong, China; 5 Department of Pathology, Queen Mary Hospital, Hong Kong, China; 6 Department of Paediatrics and Adolescent Medicine, Li Ka Shing Faculty of Medicine, The University of Hong Kong, Hong Kong, SAR China

## Abstract

**Background:**

Neutrophil is of the most abundant number in human immune system. During acute influenza virus infection, neutrophils are already active in the early phase of inflammation - a time in which clinical biopsy or autopsy material is not readily available. However, the role of neutrophil in virus infection is not well understood. Here, we studied the role of neutrophil in host defense during influenza A virus infection, specifically assessing if it contributes to the differential pathogenesis in H5N1 disease.

**Methods:**

Neutrophils were freshly isolated from healthy volunteers and subjected to direct influenza H1N1 and H5N1 virus infection in vitro. The ability of the naïve neutrophils to infiltrate from the basolateral to the apical phase of the influenza virus infected alveolar epithelium was assessed. The viral replication, innate immune responses and Neutrophil extracellular trap (NET) formation of neutrophils upon influenza virus infection were evaluated.

**Results:**

Our results demonstrated that influenza virus infected alveolar epithelium allowed neutrophil transmigration. Significantly more neutrophils migrated across the H5N1 influenza virus infected the epithelium than the counterpart infected by the seasonal influenza H1N1 virus infected. Neutrophils were equally susceptible to H5N1 and H1N1 virus infection with similar viral gene transcription. Productive replication was observed in H5N1 infected neutrophils. H5N1 induced higher cytokine and chemokine gene transcription than H1N1 infected neutrophils, including TNF-α, IFN-β, CXCL10, MIP-1α and IL-8. This inferred a more intense inflammatory response posed by H5N1 than H1N1 virus. Strikingly, NADPH oxidase-independent NET formation was only observed in H1N1 infected neutrophils at 6 hpi while no NET formation was observed upon H5N1 infection.

**Conclusion:**

Our data is the first to demonstrate that NET formation is abrogated in H5N1 influenza virus infection and might contribute to the severity of H5N1 disease.

## Background

Neutrophils account for 50–70% of circulating white blood cells and are the most abundant cells of the human innate immune system [1]. Neutrophils infiltration is detectable in the early phase of inflammation [2, 3], a time at which clinical biopsy or autopsy material is rarely available for the case of seasonal influenza infection. Studies in experimentally infected mice suggest that neutrophils secrete chemokines such as CXCL12 and contribute to the recruitment of influenza virus-specific CD8+ T cells to sites of infection [4, 5]. Depletion of neutrophils in mice infected with a sub-lethal dose of influenza virus resulted in impaired viral clearance and fatal outcome [2, 6]. In contrast, an excessive infiltration and activation of neutrophils with the release of reactive oxygen species only resulting in mild pathology [7, 8]. Since different influenza virus subtypes vary in their virulence, their direct interaction to neutrophils warrants further investigation.

In 2004, NETosis, a novel form of programmed cell death with the release of neutrophil extracellular traps (NETs) was identified [9, 10]. NETs are web-like structures composed of double-stranded DNA (dsDNA) coated with histones and antimicrobial molecules such as neutrophil elastase (NE), α-defensin and myeloperoxidase (MPO) [10]. Chromatin release can be a source of autoantigens causing tissue damage and lead to various forms of autoimmune diseases [11, 12]. Nevertheless, NETs can bind to, entrap and kill large microbes [13]. They also possess antiviral activity to eliminate viruses including human immunodeficiency virus-1 (HIV-1), hantavirus, poxvirus and respiratory syncytial virus (RSV) [14–17]. In contrast, pathogenic effect of NETs was examined in Balb/c mice infected with A/PR/8/34 (H1N1). Excessive NETs formation was found to contribute to the acute lung injury [7]. Recently, NET activity as assessed by the release of free DNA and MPO-DNA complexes in the plasma of pandemic H1N1 and H7N9 patients collected on the day of admission was reported to be a key predictive factor of disease outcome. Regardless of the subtype, higher levels of NET markers were detected in the plasma of severe cases [18]. Thus, the viral capacity to regulate the formation of NETs could be a contributing factor to the host immunopathology. A tightly regulated neutrophil response is necessary to facilitate influenza virus clearance without eliciting a hyper-induced inflammatory response.

In addition, we examined if the NET induced by the influenza is nicotinamide adenine dinucleotide phosphate (NADPH) oxidase dependent. During the discovery of the vital role of NET, it was shown that NADPH oxidase is essential. In that study, Chronic Granulomatous disease (CGD) patients, who have their NADPH oxidase function impaired, were having a poor antimicrobial activity upon the invasion of *Aspergillus fumigatus* infection in lung. Neutrophil of the CGD patients were not able to form NET following fungal, bacterial infection or phorbol 12-myristate 13-acetate (PMA) activation [19]. Reactive oxygen species (ROS) and NADPH oxidase are required for hantavirus and HIV-1 NET formation [14, 15], while the mechanism was not well-documented for rhinovirus and RSV [20, 21].

So far, NET induction in highly pathogenic avian influenza (HPAI) H5N1 infection has not been investigated. Here, we observed that more neutrophils transmigrated across H5N1 infected alveolar epithelium than H1N1. We compared the response of neutrophils following infection with highly pathogenic H5N1 or seasonal H1N1 influenza virus infection in vitro. NET formation was found in H1N1 infected neutrophils but not in H5N1 infected neutrophils and demonstrated that influenza-induced NET formation was NADPH oxidase-independent.

## Methods

### Viruses

A seasonal influenza A H1N1 virus (A/Oklahoma/447/08), a pandemic H1N1/09 virus (A/Hong Kong/415742/09, H1N1pdm) and two HPAI H5N1 viruses isolated from patients with fatal human H5N1 disease in Hong Kong in 1997 (A/Hong Kong/483/97) and in Vietnam in 2004 (A/Vietnam/1203/04) were used. Viruses were isolated and cultured in Madin-Darby canine kidney (MDCK) cells. Virus titration was done using tissue culture infection dose 50% (TCID_50_) assay. Infections were all performed in the BSL-3 bio-containment facility at the Core Facility of Li Ka Shing Faculty of Medicine, HKU.

### Viral titration by TCID_50_ assay

MDCK cells were seeded on 96-well tissue culture plates 1 day before the viral titration assay. Cells were washed once with PBS and changed to serum-free MEM medium with 1% PS and L-1-Tosylamide-2-phenylethyl chloromethyl ketone (TPCK)-treated trypsin. Virus samples or culture supernatants were titrated in serial half-log_10_ dilutions with serum-free medium prior to the addition of the diluted virus to MDCK cell plates in quadruplicate. The highest viral dilution leading to cytopathic effect (CPE) in ~ 50% of inoculated wells was estimated using the Karber method.

### Primary human neutrophils isolation and infection

10 ml of peripheral blood was drawn from healthy volunteers aged from 24 to 40 years old and CGD patients aged from 5 to 16 years old. The collection of patients’ peripheral blood was approved by the HKU/HA HKW Institutional Review Board (UW 10–430 and 15–026).

Primary human neutrophils were isolated by density centrifugation with Histopaque 1077 and 1119. The granulocyte layer was purified from red blood cell using lysis buffer and washed with PBS as described [1]. The purified neutrophils were cultured in RPMI-1640 medium without phenol red with 10% FBS and 1% PS. The average yield of this protocol was 1.57 ± 0.21 × 10^6^ cell/ml, *n* = 5 with an approximately 20–21 h(h) lifespan. The freshly isolated neutrophils were seeded at a density of 1 × 10^5^ cell per well onto a 24-well tissue culture plates and settled for 1 h before infection experiments. Neutrophils were pre-incubated with or without 10 μM Diphenyleneiodonium chloride (DPI), an inhibitor of NADPH oxidase before influenza virus infection and the treatment continued post-infection. Thereafter, influenza virus at a multiplicity of infection (MOI) of 0.01 and 2 was added into the neutrophil cultures. For transepithelial migration experiments, 5 × 10^5^ naïve neutrophils were directly added to the apical chamber with alveolar epithelial cells as described below.

### Isolation and culture of primary human type I-like pneumocytes

Primary human type I-like pneumocytes were isolated from the lung tissue obtained from patients underwent lung resection in the Department of Cardiothoracic Surgery, Queen Mary Hospital, Hong Kong SAR, with the approval of Institutional Review Board of the University of Hong Kong and Hospital Authority Hong Kong West Cluster (UW 10–430). Pneumocytes were prepared as previously described [22]. Briefly, lung tissue was chopped into pieces of 0.5 mm thickness using tissue chopper and digested using a combination of trypsin and elastase for 45 min at 37 °C in a shaking water-bath. The cell population was purified by cell attachment, percoll density gradient centrifugation and magnetic cell sorting. The cells were maintained in a humidified atmosphere (5% CO_2_, 37 °C) under liquid-covered conditions, and small airway growth medium, SAGM, (Lonza, USA) was used and changed every 48 h starting from 48 h after cell plating.

### Neutrophil Transepithelial migration assay

**Fig. 1:**
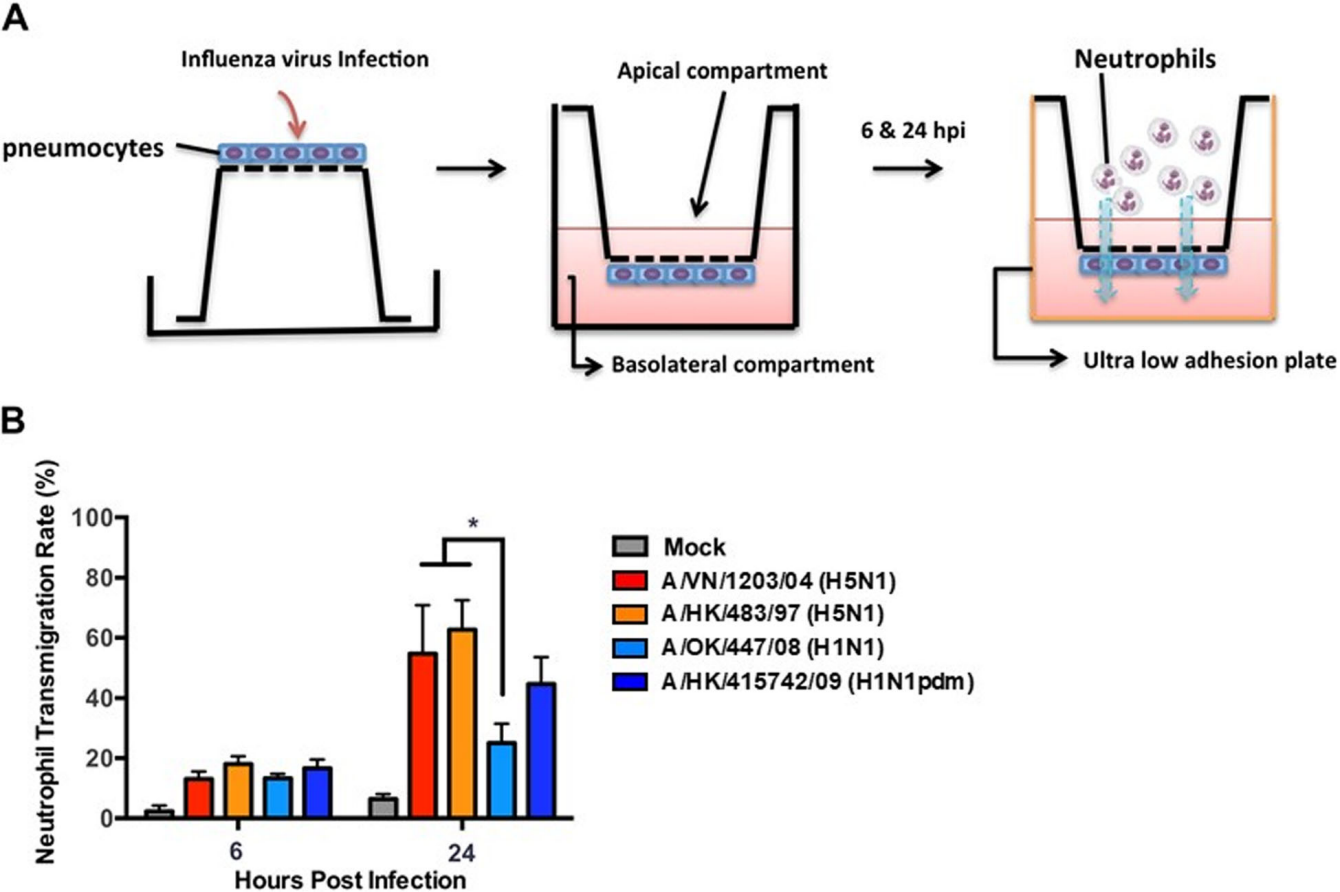
Neutrophil transepithelial migration in a primary human type I-like pneumocyte transwell system. **a** Schematic diagram showing the setup of transepithelial migration. Pneumocytes (Pϕ), indicated as the blue cells, were seeded at the basolateral side of the transwell membrane with a pore size of 3.0 μm. Naïve neutrophils (Nϕ) were added at the apical chamber, as if they infiltrate from the basolateral size of Pϕ to the alveolar space (basolateral chamber) in vivo. **b** Percentage of neutrophil migration across mock (grey) and influenza virus infected Pϕ layer at 6 and 24 hpi. * indicates *p* < 0.05 between the indicated group(s)

3 × 10^5^ pneumocytes were seeded on the basolateral side of a transwell insert with a 3.0 μm pore size membrane by inverting the transwells (Fig. 1a). The setup was incubated at a 37 °C, 5% CO_2_ incubator for 6 h to allow cell attachment. After 6 h, the transwells were flipped upright and medium was added back to both apical and basolateral chambers and the monolayer was allowed to grow for at least 48 h into a tight monolayer, confirmed by transepithelial resistance measurements.

To infect the pneumocytes, transwells were inverted with the apical surface of the pneumocytes exposed to 80 μl of influenza virus at a multiplicity of infection (MOI) of 0.01 for 1 h followed by PBS wash for three times. The transwells were then returned to its original orientation in a 24 well ultra-low adhesion plate with small airway growth medium (SAGM, Lonza). At 6 and 24 hpi, 5 × 10^5^ naïve neutrophils were added to the apical chamber, and 90 mins were allowed for the transepithelial migration to take place [23]. After 90 mins, the number of neutrophils that migrated though the epithelial monolayer into the lower chamber was counted and expressed in percentage of transmigration.

### Influenza viral gene, protein expression and infectious titer determination

Virus transcription was assessed by influenza matrix (M) gene copy numbers in RNA lysate of the infected cells at 1, 3, 6 and 16 hpi. Replicate cultures of infected neutrophils on coverslips were fixed with 4% paraformaldehyde (PFA) at 6 hpi for immunofluorescence assay using FITC-conjugated antibodies against influenza nucleoprotein and matrix protein. The supernatant samples of the infected cultures were collected for viral titration assay in MDCK cell in 96 well-plate format and expressed in TCID_50_/ml.

### Immunofluorescence staining for influenza antigen

The neutrophils seeded on coverslips were fixed with 4% paraformaldehyde (PFA) at 6 hpi. These cells were stained with mouse anti-influenza virus nucleoprotiein (NP) and matrix (M) protein antibodies conjugated with fluorescein isothiocyanate (FITC) (Dako Diagnostics Ltd., Ely, United Kingdom). After staining, the coverslips were mounted onto glass slides using a mounting medium with 4′, 6-diamidino-2-phenylindole (DAPI) (Vectashield mounting medium with DAPI; Vector Laboratories Inc.).

### Thermal inactivation of virus

In order to have an objective measure of the overall viral load in the experimental setup, including those infectious viruses that were thermally inactivated at 37 °C during the experiment period, thermal inactivation measurements of influenza virus of the two subtypes at a gradient of concentrations was performed. 1 ml of viruses at different concentrations (10^2^–10^6^ TCID_50_/ml) was put in a 24-well plate and placed in the 37 °C incubator. 130 ul of the virus were collected at 1, 6, and 16 h post incubation along the experimental duration of our in vitro study. The virus titer of these samples was measured by the viral titration and thus the thermal inactivation curves of these viruses at 37 °C were constructed. These curves were used to plot along with the viral titer of the virus inoculated neutrophil culture collected at the same time point.

### Cytokine and chemokine gene expression in naïve neutrophils after influenza virus infection

At 6 and 16 hpi, the neutrophils infected with different strains of influenza viruses were lysed using RLT and beta-mercaptoethanol and mRNA of cells were extracted using QIAGEN RNAeasy Kit following manufacturer’s instruction. Reverse transcription was carried out with a PrimeScript RT reagent kit (Takara Biotechnology, Dalian, China) The quantitative PCR (qPCR) SYBR Premix Ex Taq (Takara Biotechnology, Dalian, China) was used and the real-time qPCR assays were run on a ViiA™ 7 Real Time PCR System (Life technology, Carlsbad, CA). Viral M gene expression, host cytokines (TNF-α, IFN-β) and chemokines (CXCL10, CCL2, IL-8 and MIP-1 α) gene expression and the housekeeping (β-actin) gene expression in absolute copy numbers were determined using a standard curve generated by a serial dilution of a standard plasmid with known copy numbers, which was included in the qPCR simultaneously. These gene expressions were normalized by using the housekeeping gene product β-actin mRNA and expressed as copy number per 10^5^ β-actin expressions.

### Measurement of NET formation

NET formation was visualized with immunofluorescence staining with 5 μM of Sytox Green (Invitrogen, California, United States) before fixation 4% PFA. Coverslips were mounted onto glass slides with VECTASHIELD® Antifade Mounting Medium with DAPI.

To quantitatively assess the level of NET formation induced by the different strains of influenza virus, neutrophils (1 × 10^4^ cell/well) were seeded into a sterile 96-well flat-bottom black plate. At 6 hpi, the cells were stained with 5 μM Sytox Green for 5 mins and relative fluorescence intensity was determined using a FLUOstar OPTIMA microplate reader (BMG Labtech, Ortenberg, Germany) at an excitation wavelength of 485 nm and an emission wavelength of 520 nm. The relative fluorescence units (RFU) of infected cells were obtained after subtracting the fluorescence of unstimulated neutrophils. The RFU of the mock-infected neutrophils was set as 100%. The plotted RFU of neutrophils infected with influenza viruses was calculated by dividing the raw RFU reading by that of mock-infected neutrophils.

### Scanning electron microscopy (SEM)

To observe the formation of NET, control and infected neutrophils were fixed in 2.5% glutaraldehyde in 0.1 M sodium cacodylate-HCL buffer pH 7.4 at 1, 6 and 21hpi. Fixed cells on coverslip were washed in cacodylate buffer with 0.1 M sucrose to remove excess fixative and post-fix in osmium tetroxide for 1 h at room temperature. The cells were then dehydrated using increased concentration of ethanol each for 15 mins from 30, 50, 70, 90, to 100%. The cells were dried in a critical point dryer using liquid carbon dioxide as transitional fluid and the coverslips were then mount on the specimen holders. A thin layer (100-200 Å) of metallic film was coated on the specimen surface using a vacuum evaporator. The samples were kept in a desiccator until viewing. Hitachi S-4800 FEG scanning electron microscope (Electron Microscope Unit, HKU) was used to examine these samples, with the EMAX energy software.

### Statistical analysis

Mock-infected cells served as negative controls and each experiment was repeated at least three times using cells from different donors and the dataset were pooled. The mean and standard error of mean (SEM) of three experiments were shown. The gene expression of different cytokines and chemokines was compared among different treatments at each time point using one-way ANOVA. *Bonferroni* multiple comparison test was used as the *post* test to determine significance between treatments. *P* value less than 0.05 was deemed to be statistically significant. Statistical analyses were done with GraphPad Prism 6.0 (GraphPad Software, La Jolla, CA, USA).

## Results

### Neutrophils transmigrate through the alveolar epithelium upon influenza virus infection

In order to examine if differential neutrophils transmigration upon H5N1 and H1N1 infection would take place, we developed an in vitro model to quantify the number of neutrophils transmigrated across alveolar epithelium using transwell inserts. At 6 hpi, the transepithelial migration of neutrophils across the alveolar epithelia infected by all influenza subtypes were all under 20% (Fig. 1b). At 24hpi, A/VN1203/04 (H5N1) and A/HK/483/97 (H5N1)-infected alveolar epithelia allowed significantly greater number of neutrophil transmigration than that of seasonal A/HK/54/98 (H1N1)-infected alveolar epithelium. The transepithelial migration rate of neutrophil in A/HK/415742/09 (H1N1pdm) infected epithelium was intermediate between H5N1 and seasonal H1N1 viruses and was not statistically different to either. As a negative control, mock-infected epithelium resulted in a transmigration rate of less than 6%.

### Naïve neutrophils are susceptible to influenza virus infection

**Fig. 2:**
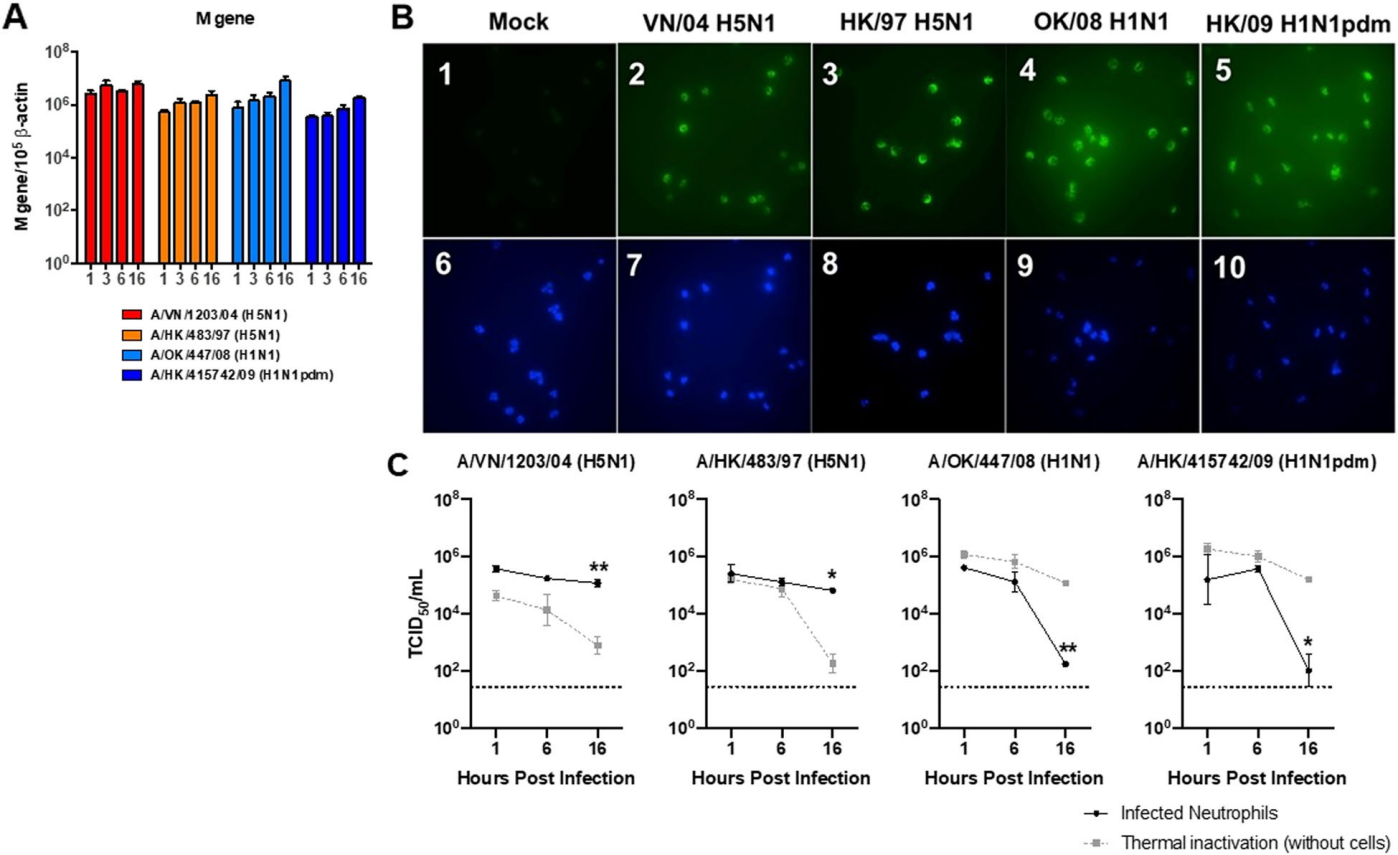
Influenza virus infection and replication competence in naïve neutrophils. Primary human neutrophils isolated freshly from healthy donor were infected with influenza viruses A/VN/1203/04 (H5N1), A/HK/483/97 (H5N1), A/OK/447/08 (H1N1) and A/HK/415742/09 (H1N1pdm) at a MOI of 2. The bar charts show the means and the standard error of mean of the virus quantities pooled from at least three independent experiments. (**a**) Influenza matrix gene expression in these infected neutrophils at 1, 3, 6, and 16 hpi were plotted. (**b**) At 16 hpi, the infected neutrophils were fixed in 4% PFA and stained for influenza nucleoprotein and matrix protein (Green, 1–5) with a counterstain of DNA material using DAPI (Blue, 6–10). (**c**) Viral load in the neutrophil cultures were monitored at 1, 6, and 16 hpi using viral titration assay (black lines). Thermal inactivation kinetics of the same virus strain without cells at 10^6^ TCID_50_/ml was plotted for comparison (grey dashed lines). Horizontal dotted line denotes detection limit of TCID_50_ assay. Key: *, *p* < 0.05 and **, *p* < 0.01 significant differences of viral load across the indicated group(s)

Upon the infiltration of neutrophils into the alveoli, the neutrophils would expose to influenza virus. We performed an infection experiment to examine if the influenza virus can infect and replicate in neutrophils. Influenza M gene was readily detected at 1 hpi with copy numbers of 10^6^ for both subtypes of influenza virus, H5N1 and H1N1. There was no increasing trend of viral gene copies in the cells at later time points from 3, 6 to 16 hpi (Fig. 2a). We investigated for de novo viral protein by immunofluorescent assay at 16 hpi and found evidence of viral antigen in the multi-lobed nuclei of neutrophils infected with each of the four viruses while mock-infected cells remained unstained. This finding suggested that viral protein translation had taken place in both H5N1 and H1N1 infected neutrophils (Fig. 2b). To examine if infectious virions were formed, we titrated the culture supernatant collected at different time points post infection (Fig. 2c). A thermal inactivation control of H5N1, seasonal and pandemic H1N1 were included for better comparison to indicate the viral load degradation at 37 °C along the experiment time course without the presence of susceptible cells as indicated by grey dashed lines. Infectious virus titers in the culture supernatant of the H5N1 infected cells at 16 hpi was significantly higher than that in the thermal inactivation controls while those of the seasonal and pandemic H1N1 infected cultures were significantly lower than the thermal inactivation controls. This suggested that H5N1 viruses were productively replicating in H5N1 infected neutrophils whereas H1N1 viruses were being actively inactivated by neutrophils (Fig. 2c).

### Cytokine and chemokine gene expression in naïve neutrophils

**Fig. 3:**
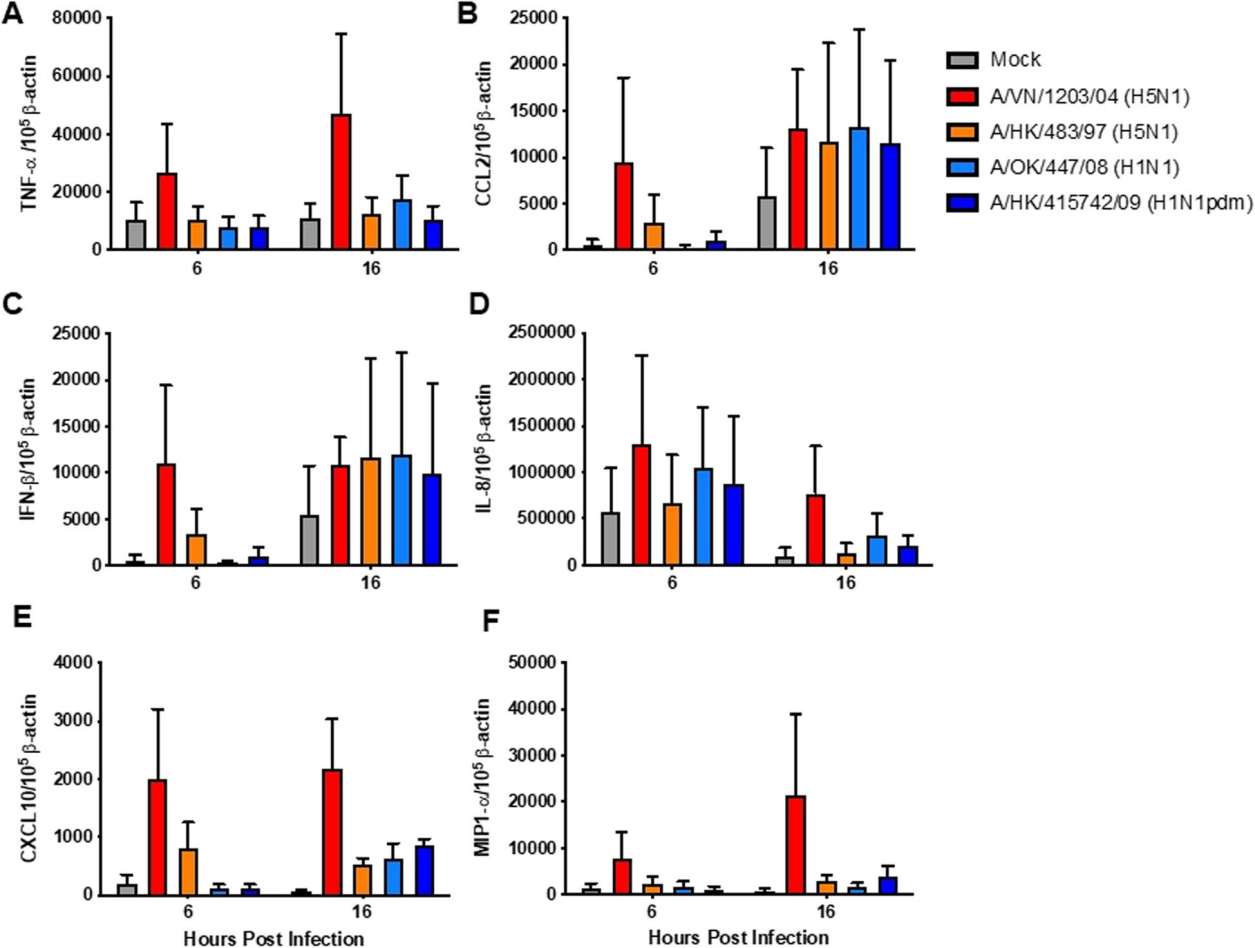
Cytokine and chemokine expression in influenza virus infected neutrophil at 6 and 16hpi. Neutrophils were infected with the influenza viruses at a MOI of 2. The graph shows the means and SEM of mRNA copies of (**a**) TNF-α, (**b**) CCL2, (C) IFN-β, (**d**) IL-8, (**e**) CXCL10, and (**f**) MIP1-α expressed per 10^5^ β-actin copies from four representative experiments

We investigated whether the differential viral load detected in the infected neutrophil cultures was correlated with the innate immune response of the neutrophils. We found a trend (although not statistically significant) of higher mRNA expression of TNF-α (Fig. 3a), IFN-β (Fig. 3c), IL-8 (Fig. 3d), CXCL10 (Fig. 3e) and MIP-1α (Fig. 3f) detected in H5N1 infected neutrophils (red and orange bars) compared with that of H1N1 and H1N1pdm (blue bars) at both 6 and 16 hpi.

### Low pathogenic seasonal H1N1 virus but not highly pathogenic H5N1 induces neutrophil extracellular traps (NETs)

**Fig. 4:**
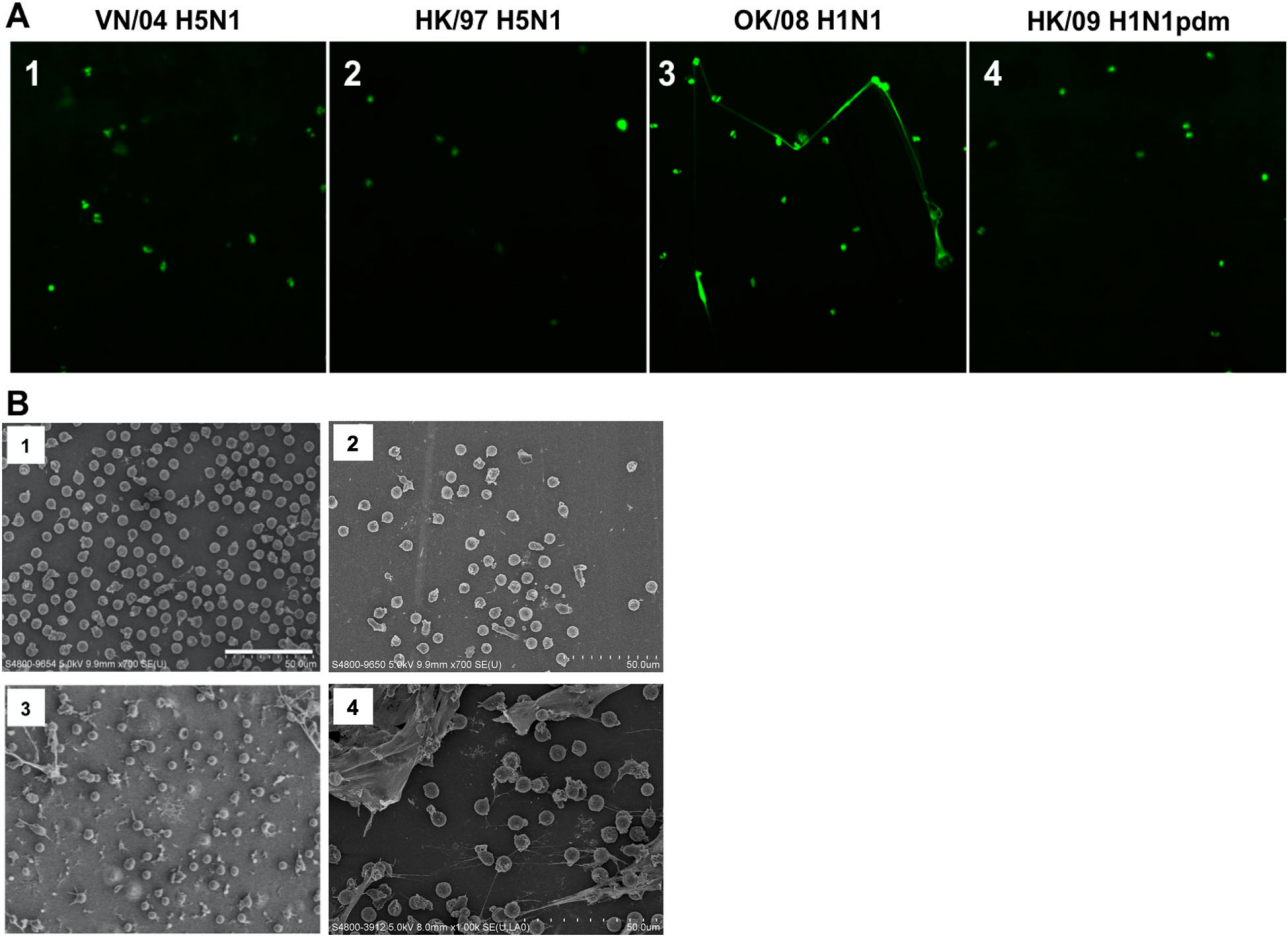
Neutrophil extracellular trap formation upon influenza virus infection with and without pre-treatment of virus-free condition media. **a** SYTOX green staining for the extracellular DNA in [1] A/VN/1203/04 (H5N1), [2] A/HK/483/97 (H5N1), [3] A/OK/447/08 (H1N1) and [4] A/HK/415742/09 (H1N1pdm) infected neutrophil at 6 hpi. The A/OK/447/08 (H1N1) infected neutrophil showed the entrenched morphology of the staining represents stretching out of DNA material from the nucleus of neutrophil which is one of the typical indicators of NET formation. **b** Scanning electron microscopy of [1] mock, [2] A/HK/483/97 (H5N1) [3] A/OK/447/08 (H1N1) infected neutrophils at 6 hpi, scale bar = 50 μm

With the use of SYTOX green staining, web-like structures (NETs) were observed extensively in seasonal H1N1 induced neutrophils and less so in the H1N1pdm infected neutrophils, with the extracellular dsDNA was stained in green in the live neutrophils (Fig. 4a-3). In contrast, NET formation was not seen in the H5N1 or mock-infected cells (Fig. 4a-1 and a-2). The results were validated using scanning electron microscopy (Fig. 4b). The mock and H5N1-infected neutrophil did not induce the formation of NET (Fig. 4b-1 and 2) while the H1N1 (Fig. 4b-3) and the H1N1pdm (Fig. 4b-4) induced the formation of NET to different extents.

### NADPH oxidase is not required for NET generation in influenza virus infection

**Fig. 5:**
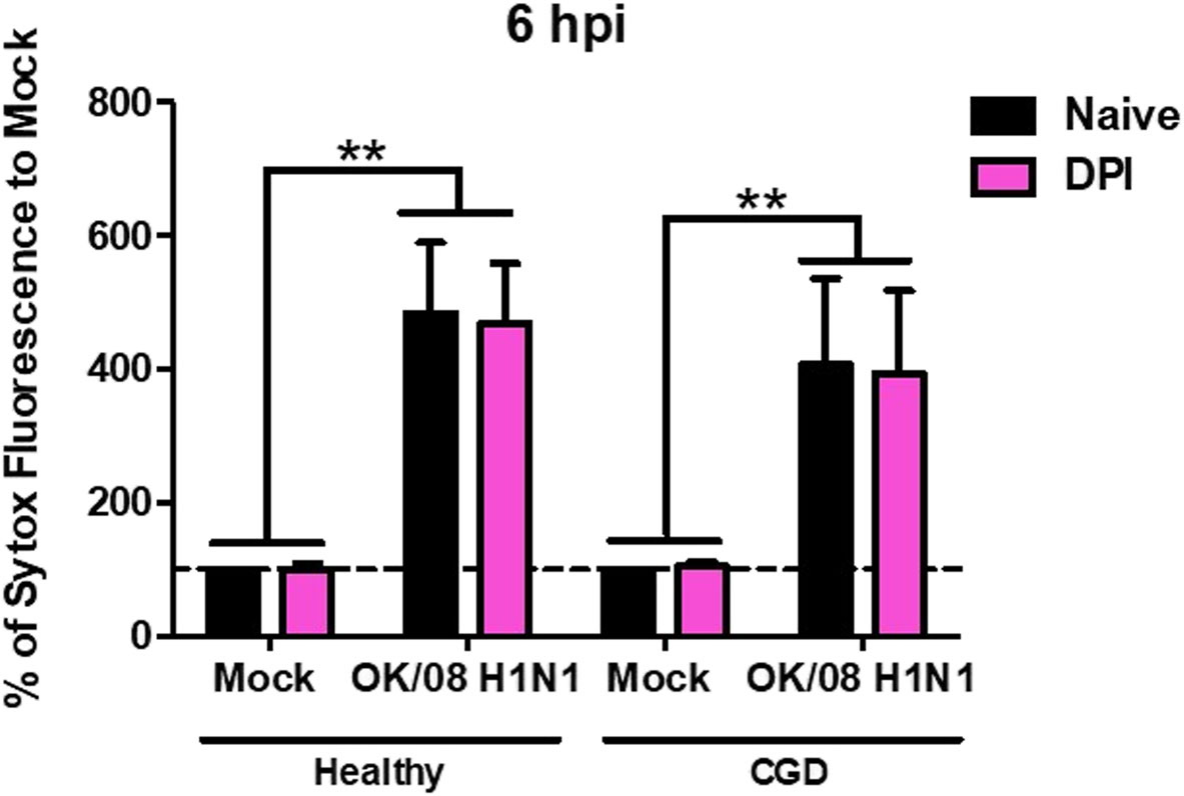
Neutrophil extracellular trap formation of neutrophils isolated from healthy donors and chronic granulomatous disease (CGD) patients with and without DPI treatment. Relative fluorescence intensity of SYTOX green after infection was measured by the FLUOstar OPTIMA microplate reader at 6 hpi. The relative fluorescence unit (RFU) of the mock-infected neutrophils was set as 100%. Key: **, *p* < 0.01 indicates significant difference of the percentage of NET formation of neutrophils among group(s)

In order to understand the pathway activated by that influenza virus to induce the formation of NET, we compared the ability of NET formation upon H1N1 influenza virus challenge in the neutrophils isolated from healthy donors and CGD patients. Interestingly, H1N1 infection could stimulate NET formation in CGD patients’ neutrophils (Fig. 5).

To specifically confirm that NADPH oxidase was not involved in H1N1 influenza virus induced NET formation, we performed an additional experiment in the presence of a NADPH oxidase inhibitor, diphenyleneiodonium (DPI). DPI treatment did not inhibit NETs induced by H1N1 infection in neutrophils from the healthy donors and the CGD patients. This implies that NETosis stimulated by H1N1 influenza virus infection is via a mechanism distinct from that induced by PMA or bacteria where NET formation is NADPH oxidase dependent.

## Discussion

Neutrophil is the first immune cell population recruited to sites of infection and neutrophil infiltration is a characteristic feature in the early phase of influenza virus infection in humans, ferrets and mice [2, 24–26]. In our in vitro neutrophil transepithelial migration model, H5N1-infection of alveolar epithelia resulted in a transmigration rate of 55 to 60%, which was significantly greater than that of H1N1 and H1N1pdm at 24 hpi. Our data is consistent with an animal study using Balb/c mice infected with A/Thailand/16/2004 (H5N1) and A/South Carolina/1/18 virus (H1N1pdm), in which influenza virus infection significantly increased the recruitment of neutrophils and macrophages at day 3 post infection at which time the lung viral titers peaked [24].

Neutrophils recruitment to the airways and lungs of infected animals was often contributed by the elevated levels of proinflammatory cytokines [6]. H5N1 elicited robust inflammatory responses [26, 27] concurrently with the greater nuclear translocation of NF-κB subunits p50 and p65 in H5N1-infected alveolar epithelial cells (Supp Fig. 1) [28, 29] and we found that H5N1 resulted in higher neutrophils transepithelial migration than H1N1.

After neutrophils reaching the alveoli, they exposed to infectious virus which buds from the apical surface of the infected alveolar epithelial cells. The susceptibility and the response of neutrophils to the virus was investigated. H5N1 viral proteins and mRNA were present in neutrophils of H5N1 infected patients and neutrophils were proposed to be a “vehicle” for viral replication and dissemination in human H5N1 infection [30]. Although influenza sialic acid receptors are lacking on the neutrophils’ surface [31], we found that neutrophils are susceptible to infection by both H1N1 or H5N1 viruses although productive infectious virus was only produced after H5N1 infection. Viral gene detected in influenza virus inoculated neutrophils might be inconclusive as could be contributed by the phagocytosed viruses. To ensure the replication cycle had taken place, the detection of H1N1 and H5N1 viral protein in the nucleus (rather than diffusely in the cytoplasm) is suggestive of de novo viral protein synthesis. Protein derived from phagocytosed virus would be present in the cytoplasm but not in the nucleus. The rapid infection and the absence of influenza virus receptors in neutrophils may imply that the way of virus entry is diverged from traditional virus binding and endocytosis. Since phagocytosis of foreign pathogens is the primary function of neutrophils, influenza viruses may enter neutrophils using this approach [32, 33].

Sensing of influenza viruses can be achieved by pattern recognition receptors (PRRs) such as Toll-like receptor (TLR)-7/8 present on neutrophils for their activation [34]. Neutrophils are known to secrete cytokines and chemokines in response to infection [34, 35] neutrophils infected with H5N1 subtypes induced a trend of stronger gene transcription of TNF-α, IFN-β, CXCL10 and MIP-1α than that of H1N1 subtypes. Since both H1N1 and H5N1 viruses infected neutrophils, it is not surprising that they all elicited cytokine responses.

We confirmed that NETs are stimulated in H1N1 infection. To further investigate if influenza virus induced NET would require NADPH oxidase, neutrophil isolated from CGD patients was employed [36] in addition to the use of a NADPH oxidase inhibitor (DPI), which serves as an alternative treatment to inhibit NETs [37]. We found that neutrophils from CGD patients’ and neutrophils treated with DPI were able to generate NETs after H1N1 stimulation. Our data is thus consistent with a study reporting that DPI treatment did not alter NET formation to influenza A virus with and without LL-37 incubation [38] and revealed that NADPH oxidase is not required for influenza virus-induced NETs. It was previously suggested that there are multiple mechanisms for NET formation than the requirements for oxidant generation dependent on stimulus, such as influx of calcium ions induced by ionomycin [39].

Surprisingly, we found that H5N1 viruses fail to stimulate NETosis. The mechanisms of how H5N1 viruses impair the triggering NETosis would be important to investigate. The pathogenic significance of this difference in relation to the severe disease caused by H5N1 viruses should be further investigated.

There were several limitations needed to be addressed in this study. With the focus in understanding the direct interaction between neutrophil and influenza virus, we did not study comprehensively the effect of infected alveolar epithelial cells on the neutrophils after influenza virus infection. Moreover, due to the operation safety in the BSL-3 setting, we only measured the host response at the gene level. Lastly, in terms of the mechanistic investigation, we ruled out the involvement of NADPH oxidase dependence in the mechanistic pathway of H1N1 induced NET formation and did not further investigate other possible pathways.

## Conclusion

To conclude, more neutrophils could transmigrate across H5N1 influenza virus infected alveolar epithelium than the H1N1 infected epithelium. We revealed that both H1N1 and H5N1 viruses was able to infect in neutrophils but only H5N1 yielded infectious virus. H5N1 viruses also elicited higher cytokine and chemokine expressions than H1N1 infection. NET formation was seen only in H1N1 infection and may have provided an antiviral effect which was lacking in H5N1 infections. Apart from the dysregulation of cytokines and chemokines and the acute lung injury caused by excessive viral replication, the inability of H5N1 to stimulate NETs could be a mechanism underlying the differential pathogenesis of influenza diseases. The mechanism of influenza virus-induced NET and their functional roles, for example its role in limiting the viral load, are yet to be elucidated.

## Supplementary information

**Additional file 1 Fig. S1.** p50 and p65 nuclear translocation in human type I-like pneumocyte upon influenza virus infection

## Data Availability

The authors confirm that the data supporting the findings of this study are available within the article and its supplementary materials.

## Abbreviations

BSL-3: Biosafety Level 3
CGD: Chronic granulomatous disease
DPI: Diphenyleneiodonium chloride
HIV-1: Human immunodeficiency virus-1
ROS: Reactive oxygen species
RSV: Respiratory syncytial virus
TLR: Toll like receptor
MPO: Myeloperoxidase
NADPH: Nicotinamide adenine dinucleotide phosphate
NE: Neutrophil elastase
NET: Neutrophil extracellular trap
PMA: Phorbol myristate acetate
PRRs: Pattern recognition receptors

## References

[1] Borregaard N (2010). Neutrophils, from marrow to microbes. Immunity..

[2] Tate MD, Deng YM, Jones JE, Anderson GP, Brooks AG, Reading PC (2009). Neutrophils ameliorate lung injury and the development of severe disease during influenza infection. J Immunol.

[3] White MR, Tecle T, Crouch EC, Hartshorn KL (2007). Impact of neutrophils on antiviral activity of human bronchoalveolar lavage fluid. Am J Phys Lung Cell Mol Phys.

[4] Lim K, Hyun YM, Lambert-Emo K, Capece T, Bae S, Miller R (2015). Neutrophil trails guide influenza-specific CD8(+) T cells in the airways. Science.

[5] Tate MD, Brooks AG, Reading PC, Mintern JD (2012). Neutrophils sustain effective CD8(+) T-cell responses in the respiratory tract following influenza infection. Immunol Cell Biol.

[6] Tate MD, Ioannidis LJ, Croker B, Brown LE, Brooks AG, Reading PC (2011). The role of neutrophils during mild and severe influenza virus infections of mice. PLoS One.

[7] Narasaraju T, Yang E, Samy RP, Ng HH, Poh WP, Liew AA (2011). Excessive neutrophils and neutrophil extracellular traps contribute to acute lung injury of influenza pneumonitis. Am J Pathol.

[8] Garcia CC, Tavares LP, Dias ACF, Kehdy F, Alvarado-Arnez LE, Queiroz-Junior CM (2018). Phosphatidyl inositol 3 kinase-gamma balances antiviral and inflammatory responses during influenza a H1N1 infection: from murine model to genetic association in patients. Front Immunol.

[9] Takei H, Araki A, Watanabe H, Ichinose A, Sendo F (1996). Rapid killing of human neutrophils by the potent activator phorbol 12-myristate 13-acetate (PMA) accompanied by changes different from typical apoptosis or necrosis. J Leukoc Biol.

[10] Brinkmann V, Reichard U, Goosmann C, Fauler B, Uhlemann Y, Weiss DS (2004). Neutrophil extracellular traps kill bacteria. Science..

[11] Khandpur R, Carmona-Rivera C, Vivekanandan-Giri A, Gizinski A, Yalavarthi S, Knight JS (2013). NETs are a source of citrullinated autoantigens and stimulate inflammatory responses in rheumatoid arthritis. Sci Transl Med.

[12] Lande R, Ganguly D, Facchinetti V, Frasca L, Conrad C, Gregorio J (2011). Neutrophils activate plasmacytoid dendritic cells by releasing self-DNA-peptide complexes in systemic lupus erythematosus. Sci Transl Med.

[13] Fuchs TA, Abed U, Goosmann C, Hurwitz R, Schulze I, Wahn V (2007). Novel cell death program leads to neutrophil extracellular traps. J Cell Biol.

[14] Raftery MJ, Lalwani P, Krautkrmer E, Peters T, Scharffetter-Kochanek K, Kruger R (2014). beta2 integrin mediates hantavirus-induced release of neutrophil extracellular traps. J Exp Med.

[15] Saitoh T, Komano J, Saitoh Y, Misawa T, Takahama M, Kozaki T (2012). Neutrophil extracellular traps mediate a host defense response to human immunodeficiency virus-1. Cell Host Microbe.

[16] Jenne CN, Wong CH, Zemp FJ, McDonald B, Rahman MM, Forsyth PA (2013). Neutrophils recruited to sites of infection protect from virus challenge by releasing neutrophil extracellular traps. Cell Host Microbe.

[17] Souza PSS, Barbosa LV, Diniz LFA, da Silva GS, Lopes BRP, Souza PMR (2018). Neutrophil extracellular traps possess anti-human respiratory syncytial virus activity: possible interaction with the viral F protein. Virus Res.

[18] Zhu L, Liu L, Zhang Y, Pu L, Liu J, Li X (2018). High level of neutrophil extracellular traps correlates with poor prognosis of severe influenza a infection. J Infect Dis.

[19] Bianchi M, Hakkim A, Brinkmann V, Siler U, Seger RA, Zychlinsky A (2009). Restoration of NET formation by gene therapy in CGD controls aspergillosis. Blood..

[20] Cortjens B, de Boer OJ, de Jong R, Antonis AF, Sabogal Pineros YS, Lutter R (2016). Neutrophil extracellular traps cause airway obstruction during respiratory syncytial virus disease. J Pathol.

[21] Toussaint M, Jackson DJ, Swieboda D, Guedan A, Tsourouktsoglou TD, Ching YM (2017). Host DNA released by NETosis promotes rhinovirus-induced type-2 allergic asthma exacerbation. Nat Med.

[22] Chan LL, Bui CT, Mok CK, Ng MM, Nicholls JM, Peiris JS (2016). Evaluation of the human adaptation of influenza a/H7N9 virus in PB2 protein using human and swine respiratory tract explant cultures. Sci Rep.

[23] Zemans RL, Briones N, Campbell M, McClendon J, Young SK, Suzuki T (2011). Neutrophil transmigration triggers repair of the lung epithelium via beta-catenin signaling. Proc Natl Acad Sci U S A.

[24] Perrone LA, Plowden JK, Garcia-Sastre A, Katz JM, Tumpey TM (2008). H5N1 and 1918 pandemic influenza virus infection results in early and excessive infiltration of macrophages and neutrophils in the lungs of mice. PLoS Pathog.

[25] Herold S, von Wulffen W, Steinmueller M, Pleschka S, Kuziel WA, Mack M (2006). Alveolar epithelial cells direct monocyte transepithelial migration upon influenza virus infection: impact of chemokines and adhesion molecules. J Immunol.

[26] Chan MC, Cheung CY, Chui WH, Tsao SW, Nicholls JM, Chan YO (2005). Proinflammatory cytokine responses induced by influenza a (H5N1) viruses in primary human alveolar and bronchial epithelial cells. Respir Res.

[27] Yu WC, Chan RW, Wang J, Travanty EA, Nicholls JM, Peiris JS (2011). Viral replication and innate host responses in primary human alveolar epithelial cells and alveolar macrophages infected with influenza H5N1 and H1N1 viruses. J Virol.

[28] Droebner K, Reiling SJ, Planz O (2008). Role of hypercytokinemia in NF-kappaB p50-deficient mice after H5N1 influenza a virus infection. J Virol.

[29] Schmolke M, Viemann D, Roth J, Ludwig S (2009). Essential impact of NF-kappaB signaling on the H5N1 influenza a virus-induced transcriptome. J Immunol.

[30] Zhao Y, Lu M, Lau LT, Lu J, Gao Z, Liu J (2008). Neutrophils may be a vehicle for viral replication and dissemination in human H5N1 avian influenza. Clin Infect Dis.

[31] Zhang Z, Huang T, Yu F, Liu X, Zhao C, Chen X (2015). Infectious progeny of 2009 a (H1N1) influenza virus replicated in and released from human neutrophils. Sci Rep.

[32] Drescher B, Bai F (2013). Neutrophil in viral infections, friend or foe?. Virus Res.

[33] Papayannopoulos V, Zychlinsky A (2009). NETs: a new strategy for using old weapons. Trends Immunol.

[34] Wang JP, Bowen GN, Padden C, Cerny A, Finberg RW, Newburger PE (2008). Toll-like receptor-mediated activation of neutrophils by influenza a virus. Blood..

[35] Tecchio C, Micheletti A, Cassatella MA (2014). Neutrophil-derived cytokines: facts beyond expression. Front Immunol.

[36] Seger RA (2008). Modern management of chronic granulomatous disease. Br J Haematol.

[37] Li Y, Trush MA (1998). Diphenyleneiodonium, an NAD(P)H oxidase inhibitor, also potently inhibits mitochondrial reactive oxygen species production. Biochem Biophys Res Commun.

[38] Tripathi S, Verma A, Kim EJ, White MR, Hartshorn KL (2014). LL-37 modulates human neutrophil responses to influenza a virus. J Leukoc Biol.

[39] Parker H, Dragunow M, Hampton MB, Kettle AJ, Winterbourn CC (2012). Requirements for NADPH oxidase and myeloperoxidase in neutrophil extracellular trap formation differ depending on the stimulus. J Leukoc Biol.

