# Supplementary material for: Host DNA released by NETosis in neutrophils exposed to seasonal H1N1 and highly pathogenic H5N1 influenza viruses
Source: Respir Res. 2020 Jun 23;21:160. doi: 10.1186/s12931-020-01425-w (PMC7310290; doi:10.1186/s12931-020-01425-w)
Supplement: Supplementary file 1 — Additional file 1 Fig. S1. p50 and p65 nuclear translocation in human type I-like pneumocyte upon influenza virus infection [file 12931_2020_1425_MOESM1_ESM.docx]

**Supplementary materials**


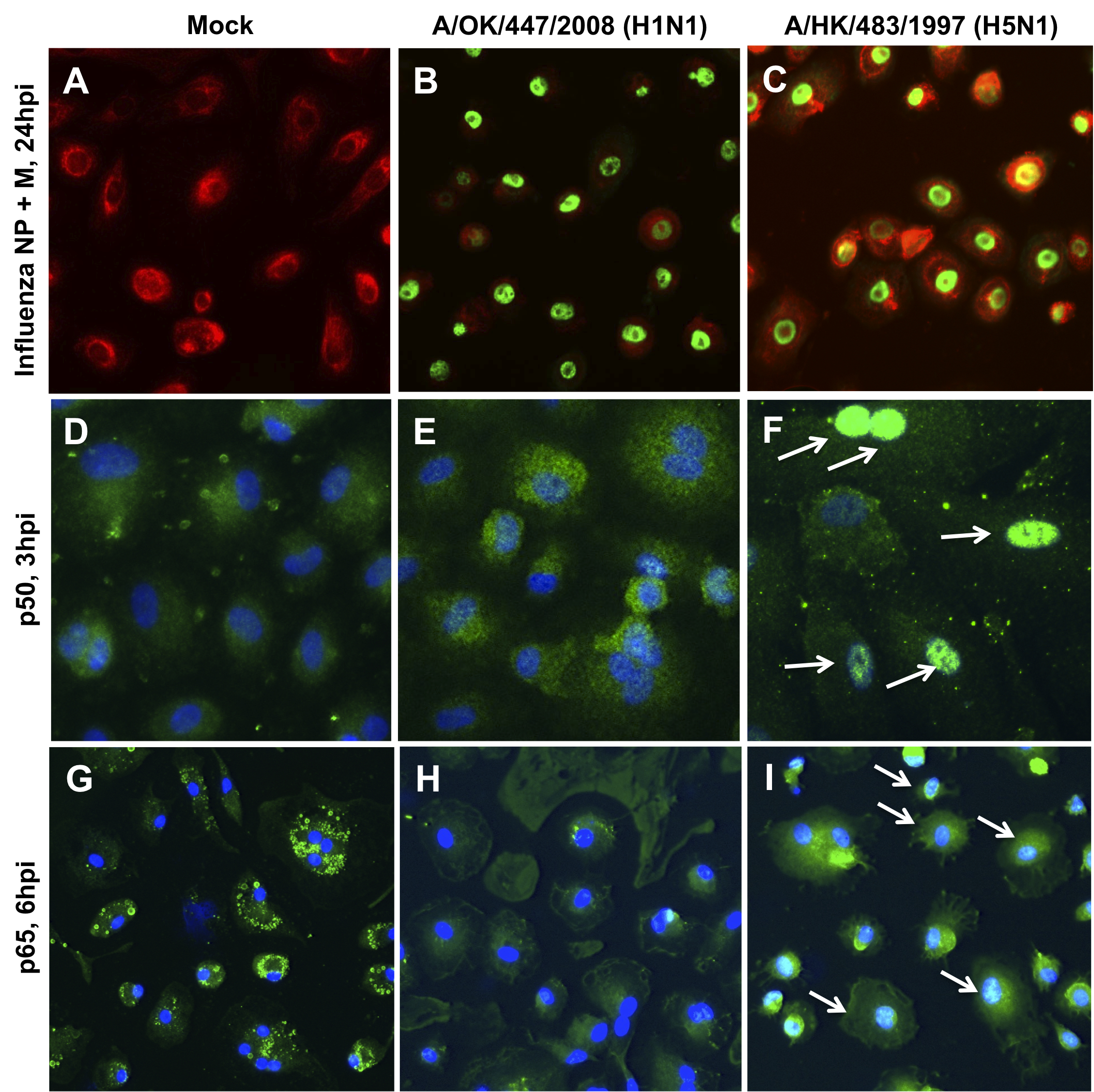


**Supp Figure 1.** **p50 and p65 nuclear translocation in human type I-like pneumocyte upon influenza virus infection.** Immunofluorescence staining of type I-like Pϕ with mock, A/OK/447/08 (H1N1) and A/HK/483/97 (H5N1). (**A-C**) The influenza virus nucleoprotein and matrix protein were stained in green with FITC-conjugated mouse antibody. At 24 hpi the infection rate of H1N1 and H5N1 was similar, as described previously(1). (**D-F**) p50 and (**G-I**) p65 proteins were stained in green with FITC-conjugated antibody, while cell nucleus DNA was stained in blue with DAPI. Nuclear translocation of these two proteins were monitored at 10 min, 20 min, 30 min, 1 h, 3 h, 6 h, 16 h and 24 h post infection with TNFα + IL-1β 1600ρg/ml incubation as a positive control. Significant p50 and p60 nuclear translocation were observed at 3 hpi and 6 hpi, respectively in H5N1 infected pneumocytes (indicate by white arrows).
